# RIMOXCLAMIN: New therapeutic regimen for Hansen’s Disease cure based on effective sensitivity recovery

**DOI:** 10.1016/j.bjid.2025.104539

**Published:** 2025-05-14

**Authors:** Marco Andrey Cipriani Frade, Gustavo Sartori Albertino, Filipe Rocha Lima, Natália Aparecida de Paula, Fabiana Aparecida Correa Cinto, Fernanda Cruz Perecin, Andrezza Westin, Wilson Marques Junior, Helena Barbosa Lugão

**Affiliations:** a Universidade de São Paulo (USP), Faculdade de Medicina de Ribeirão Preto (FMRP), Divisão de Dermatologia, Departamento de Medicina Interna, São Paulo, SP, Brazil; b Universidade de São Paulo (USP), Faculdade de Medicina de Ribeirão Preto (FMRP), Hospital das Clínicas, Centro Nacional de Referência em Dermatologia Sanitária e H.D., São Paulo, SP, Brazil; c Universidade de São Paulo (USP), Faculdade de Medicina de Ribeirão Preto (FMRP), Departamento de Bioquímica e Imunologia, São Paulo, SP, Brazil; d Universidade de São Paulo (USP), Faculdade de Medicina de Ribeirão Preto (FMRP), Hospital das Clínicas, Divisão de Assistência Farmacêutica, Unidade Especializada de Tratamento de Doenças Infeciosas (UETDI), São Paulo, SP, Brazil; e Universidade de São Paulo (USP), Faculdade de Medicina de Ribeirão Preto (FMRP), Departamento de Neurologia, Divisão de Distúrbios Neuromusculares, Ribeirão Preto, SP, Brazil

**Keywords:** Hansen’s disease, Leprosy, RIMOXCLAMIN, Treatment, Sensitivity, Multidrug therapy

## Abstract

**Background:**

World Health Organization (WHO) has recommended Multidrug Therapy (MDT/WHO) for Hansen’s Disease (HD) since 1982; nevertheless, relapse, antimicrobial resistance, and adverse reactions indicate the need for new therapeutic regimens. We evaluated the efficacy and safety of the new anti-HD regimen RIMOXCLAMIN (Rifampicin, Moxifloxacin, Clarithromycin, and Minocycline) compared with standard Multidrug Therapy provided by WHO (MDT/WHO).

**Methodology/principal findings:**

66 multibacillary HD new cases (46: RIMOXCLAMIN / 20: MDT/WHO) were evaluated between 2015 and 2023. Patients were followed up at least bimonthly by hansenologists for neurological and cutaneous findings and side effects of treatments. Hands/feet tactile sensitivity tests by Semmes Weinstein Monofilaments (SWM) and Physical Disability Grade (PDG) were carried out on the diagnosis, 3rd, 6th, and 12th months. 84.8 % and 80 % of the patients were classified as Borderline-Borderline (BB) in RIMOXCLAMIN and MDT/WHO groups, respectively, with no significant difference between them (*p* = 0.12). Nerve thickening was reduced by palpation in both groups: in RIMOXCLAMIN, reduction occurred early (65 % to 28 % at 6-months, *p* = 0.03; 9 % at 12-months, *p* = 0.03), while in MDT/WHO, it was later (95 % to 40 % at 12-months, *p* = 0.002). The greatest difference was at 6 months (*p* < 0.0001). A significant reduction was observed in pain scales on the 3rd month of treatment only with RIMOXCLAMIN; in the end, both groups showed significant reductions in pain scales, being greater in RIMOXCLAMIN group. 0.5 % reduction in the number of abnormal SWM points on the hands compared to baseline, while in the MDT/WHO group, there was an increase of abnormal points of 5.4 %. On the feet, RIMOXCLAMIN showed a reduction of 17.9 %, while in the MDT/WHO, it was 10.3 %. During follow-up, the RIMOXCLAMIN showed a significant decrease in the sum of altered SWM points compared to MDT/WHO (*p* < 0.05). Only RIMOXCLAMIN improved PDG monitoring. Both groups reported mild adverse effects.

**Conclusions/significance:**

The results indicate that RIMOXCLAMIN was superior to MDT/WHO in terms of quick recovery of neurological damage, evidenced by the improvement of symptoms and sensitivity in hands and feet as early as the third month, with a progressive improvement, maintained after the end of treatment, including a reduce of patients with PDG.

## Introduction

Hansen’s Disease (HD) is a chronic infectious disease caused by *Mycobacterium leprae* and *M. lepromatosis* with a broad spectrum of clinical manifestations.^1^^,^^2^ This bacterium mainly affects the peripheral nerves, eyes, and skin, altering skin sensation and causing muscle weakness and, consequently, disabilities and deformities.^3^

Neurological signs and symptoms are essential in active H.D. new-case finding strategies^4^, ^5^, ^6^, ^7^, ^8^ and in establishing the cardinal signs for case definition.^3^^,^^7^, ^8^, ^9^ A recent study showed that all H.D. patients diagnosed during active case-finding activities presented with dysesthesia in macular cutaneous areas, and 71.9 % had some peripheral nerve impairment.^4^ Similarly, Da Silva et al. (2021) found that all H.D. patients from a female penitentiary had dysesthesia in macular cutaneous areas, 78.6 % had abnormalities on nerve palpation (enlargement and/or pain and/or electric shock-like pain), and 71.5 % had abnormal foot tactile sensitivity detected by Semmes-Weinstein Monofilaments (SWM).^5^ Although macular presentations have been described mainly in indeterminate form, this type of lesion can be found in all clinical forms of H.D.^3^^,^^4^^,^^6^, ^7^, ^8^, ^9^, ^10^, ^11^, ^12^, ^13^ 95 % of macular H.D. patients show one or more enlarged nerves, indicating that macular cutaneous manifestations do not occur only in early H.D. cases.^6^ Additionally, it was demonstrated that the clinical finding of the typical pattern of areas with tactile sensitivity changes surrounded by normal sensation areas (“islands”), not following the territory of a specific nerve, defines the H.D. diagnosis.^6^^,^^8^

The World Health Organization (WHO) guidelines for HD recommend a multidrug regimen of rifampicin (MDT/WHO), dapsone, and clofazimine for all patients, treating with 6 monthly doses in 9 months for Paucibacillary (PB) and with 12 monthly doses in 18 months for Multibacillary (MB) HD.^14^ These three drugs have been used since 1982.

Despite its efficacy, the use of dapsone in the treatment of leprosy is associated with significant adverse effects that may compromise treatment adherence. Hemolytic anemia and methemoglobinemia are common hematological complications, particularly in patients with Glucose-6-Phosphate Dehydrogenase (G6PD) deficiency.^15^, ^16^, ^17^, ^18^ Additionally, Dapsone Hypersensitivity Syndrome (DHS), characterized by fever, skin rash, and hepatic or renal dysfunction, represents a severe and potentially fatal adverse reaction.^19^^,^^20^ Other adverse effects include neutropenia, eosinophilia, and, in rare cases, ocular toxicity such as toxic maculopathy.^15^^,^^17^^,^^21^ These complications highlight the need for safer and more effective therapeutic alternatives for leprosy treatment.

Clofazimine, another essential component of the MDT/WHO regimen, also presents significant adverse effects that impact patients’ quality of life. The most common is reddish or brownish skin discoloration, a highly visible side effect that can persist for months after treatment completion, leading to negative psychosocial consequences.^15^^,^^22^^,^^23^ Additionally, gastrointestinal toxicity, manifested as abdominal pain, nausea, and diarrhea, can further impair treatment adherence.^22^^,^^23^ In some cases, clofazimine may cause severe skin dryness and ichthyosis.^15^ Given these challenges, the search for new therapeutic regimens that minimize adverse effects while maintaining bactericidal efficacy is crucial for optimizing leprosy treatment.

Recent epidemiological data (WHO 2022) have reported that Brazil had the highest frequency of relapse after MDT/WHO, followed by India. This data and the relatively high frequencies of other re-treatment indications (WHO 2022), adverse reactions to anti-HD drugs, and detection of strains with antimicrobial resistance indicate the need for new therapeutic regimens.^22^, ^23^, ^24^ Previous studies have investigated the isolated anti-HD activity of some drugs, such as minocycline,^24^, ^25^, ^26^, ^27^ quinolones,^28^ clarithromycin,^24^^,^^25^^,^^29^ and moxifloxacin.^30^ Among these, only moxifloxacin has demonstrated superior individual bactericidal activity, indicating a promising drug to compose new treatment regimens.^30^ All these antimicrobials are described as second-line drugs for HD treatment; however, there are rare references in the literature proposing new treatment regimens using a combination of more bactericidal and effective drugs, with fewer adverse effects, against HD. Clarithromycin and minocycline may cause mild gastrointestinal symptoms, with minocycline presenting a lower risk of adverse reactions compared to other drugs.^24^^,^^25^ Their combination has shown bactericidal efficacy similar to the traditional regimen with dapsone and clofazimine, which is linked to a higher incidence of adverse effects than alternative therapies such as ROM.^27^

Our study proposes to evaluate the efficacy and safety of the new anti-HD regimen (RIMOXCLAMIN), which consists of four drugs (RIfampicin, MOXifloxacin, CLArithromycin, and MINocycline), compared with the multibacillary MDT/WHO standard regimen as an initial pilot study exploring a new and alternative proposal for a therapeutic scheme.

## Methods

### Ethics statement

This retrospective study was approved by the Research Ethics Committee at the Clinics Hospital of Ribeirão Preto Medical School, University of São Paulo (protocol n° 4.102.290 ‒ 06/22/2020). Written informed consent was obtained from every participant. All procedures involving human beings comply with the ethical standards of the Helsinki Declaration (1975/2008).

### Study design and casuistic

A comparative and retrospective study was performed from 2015 to 2023, analyzing 66 patients defined only as new cases of HD followed at the outpatient clinics at the National Referral Center in Sanitary Dermatology and HD, Hospital das Clínicas da Faculdade de Medicina de Ribeirão Preto da Universidade de São Paulo (HCFMRP-USP), Brazil, considering both public and private sectors.

### Definition of new HD cases and classification

All patients were diagnosed by clinical evaluation performed by experienced leprologists, according to the Brazilian Ministry of Health and WHO guidelines using recommended cardinal signs.^1^^,^^3^, ^4^, ^5^, ^6^, ^7^ Auxiliary tests to the clinical diagnosis were used, such as assessment of tactile sensation with a Semmes-Weinstein Monofilaments, peripheral nerves ultrasound, electroneuromyography, and other complementary exams (ELISA anti-PGL-I serology, qPCR-RLEP, and bacilloscopy), described on the medical records.^5^, ^6^, ^7^^,^^12^^,^^14^^,^^31^, ^32^, ^33^ Patients were classified considering the adapted Madrid (Congress of Madrid 1953) and the Indian Association of Leprology (IAL 1982) classifications as follows: Indeterminate (I), polar Tuberculoid (TT), Borderline-Borderline (BB), Borderline Lepromatous (BL), polar Lepromatous-Lepromatous (LL), and Pure Neural Leprosy (PNL); and PB (I and TT clinical forms) and MB (BB, BL, LL, and PNL forms) according to the WHO operational criteria. According described by Frade et al.,^5^, ^6^, ^7^ patients with whitish mild macules and with altered sensation and neurological findings were classified as borderline/multibacillary.

### Clinical neurological evaluation

The hansenologists followed up with the patients at least bimonthly, evaluating the improvement/worsening of the neurological symptoms and cutaneous signs. Muscle strength, pain intensity scales, and sensitivity mapping were carried out during the diagnosis, on the 3rd, 6th, and end of treatment.

Muscle strength was assessed using the Medical Research Council (MRC) scale, which ranges from 0 (no muscle contraction) to 5 (full muscle strength against resistance).

Pain intensity was assessed using a Numerical Rating sScale (NRS), which ranges from 0 to 10, where 0 indicates no pain, and 10 represents the worst imaginable pain. Patients were instructed to rate their pain at baseline and subsequently at each follow-up visit, allowing for the monitoring of changes in pain intensity over time.

Seven points on each hand and 11 points on each foot were tested for tactile sensation by Semmes Weinstein Monofilaments (SWM) from 0.07 *g*-force (gf) to 300gf, considering the normal skin tactile threshold as green monofilament (0.07 gf) for hands and as blue (0.2 gf) for feet, according to Brazilian Ministry of Health recommendations.^7^^,^^8^^,^^32^

Similarly, Semmes Weinstein monofilaments from 0.07 gf to 300 gf were used to test tactile sensation on hypochromic macules. When the patient felt the green monofilament, 0.07 gf,^7^ this was considered a normal skin tactile threshold.

The Physical Disability Grade (PDG) was classified as follows: Grade 0 (no H.D. patient presented PDG), Grade 1 (decreased strength and/or loss of sensation higher than 2.0 *g*-force), and Grade 2 (presence of visible disabilities and deformities).^14^^,^^21^^,^^32^

### Anti-phenolic glycolipid-I serology

Indirect ELISA was used as an index test to measure the Anti-PGL-I IgM titer of serum samples according to previously reported protocol.^31^ Serology was performed with ND-O-BSA (PGL-I) based glycoconjugate of bovine serum albumin (NR-19,346. BEI Resources). The respective index was calculated by dividing each sample's Optical Density (OD=450 nm) by the cutoff, and indices above 1.0 were considered positive.

Researchers retrospectively recorded and analyzed the first measure of ELISA Anti-PGL-I IgM levels close to diagnostic.

### Molecular diagnosis for detection of mycobacterium leprae DNA

Total DNA extraction from a skin biopsy and/or earlobes and at least one elbow, knee, and/or lesion slit-skin smear sample was performed with the QIAamp DNA Mini Kit (Qiagen, Germantown, MD, cat: 51,306) according to the manufacturer's protocol. DNA was used to perform quantitative PCR-RLEP according to a previously reported protocol.^31^ The quantitative PCR (qPCR) result was considered positive for the detection of *M. leprae* DNA with amplification up to a 40.0 Cycle threshold (Ct) and melting temperature at 87.5 °C. The maximum number of cycles used was 45.0.^33^

### Bacilloscopy

The Slit Skin Smears (SSS) remains only the laboratorial cardinal sign of HD diagnosis and are taken from four routine sites of dermal scraping samples from earlobes and at least one elbow and/or typical skin lesion. Bacilloscopy collection, analysis, and interpretation of results were carried out in accordance with Ministry of Health guidelines.^31^^,^^32^

### Peripheral nerve ultrasonography (USG)

Peripheral nerves were scanned on crosswise and lengthwise sections using portable ultrasound devices equipped with high-frequency transducers, such as Samsung HM70-A and Samsung HM70-EVO. Cross-Sectional Areas (CSA) were measured in the median (carpal tunnel and distal forearm), the ulnar (cubital tunnel and distal arm), the common fibular nerves (head of the fibula and distal thigh), and the tibial nerves (posterior to the ankles), as published before.^34^, ^35^, ^36^, ^37^, ^38^, ^39^, ^40^, ^41^

### Neurophysiologic studies

Nerve conduction studies were performed per the standard protocol described by Tomaselli et al. (2022). Compound muscle action potentials were recorded for the median, ulnar, peroneal, and tibial nerves, while Sensory Nerve Action Potentials (SNAP) were assessed for the median, ulnar, radial, sural, and superficial peroneal nerves. According to clinical evaluation, other nerves were included in some cases, such as the saphenous, plantar medial and lateral, dorsal cutaneous branch of the ulnar nerve, and medial and lateral cutaneous from the forearm. The neurophysiological pattern was defined as: (i) Asymmetrical sensory-motor neuropathy with focal slowing of the conduction velocity; (ii) Sensory-motor mononeuropathy with focal slowing of conduction velocity; (iii) Axonal sensory mononeuropathy; (iv) Asymmetrical sensory-motor axonal neuropathy; or (v) Asymmetrical sensory axonal neuropathy.^39^, ^40^, ^41^

### Definition of therapeutic groups

#### RIMOXCLAMIN multidrug therapy

A group of 46 patients were treated with the RIMOXCLAMIN multidrug therapy regimen as described in Table 1.

**Table 1:** Schematic table of RIMOXCLAMIN multidrug therapy with monthly and daily doses for one year of follow-up.

	Doses	1st and 2nd months		3rd to 12th month	
**RIMOXCLAMIN**	Monthly	RIFAMPICIN	600 mg	RIFAMPICIN	600 mg
		MOXIFLOXACIN	400 mg	MOXIFLOXACIN	400 mg
		CLARITHROMYCIN	500 mg	CLARITHROMYCIN	500 mg
				MINOCYCLINE	100 mg
	Daily	MOXIFLOXACIN	400mg	CLARITHROMYCIN	500 mg
		CLARITHROMYCIN	500mg	MINOCYCLINE	100 mg

Considering that only rifampicin is a bactericidal drug in the MDT/WHO regimen while dapsone and clofazimine are bacteriostatic ones, we sought to propose a regimen also with 12 doses of rifampicin monthly, and innovating with more bactericidal drugs such as moxifloxacin and clarithromycin in daily doses for initial two months. From the third month onwards, we decided to switch moxifloxacin to a monthly dose due to reports of tendinopathies, increased risk of aneurysm rupture, and warning of permanent adverse effects due to the use of fluoroquinolones given by the European Medicines Agency (EMA) in November 2018.^42^ Furthermore, we based ourselves on the work of Pardillo FE with auspicious results even with weekly doses using moxifloxacin, in addition to demonstrating greater antimicrobial efficacy compared to rifampicin.^30^ We then introduced minocycline to maintain the use of at least two effective antimycobacterial drugs, such as clarithromycin associated with minocycline daily as proposed, avoid treating mycobacteria with at least two effective drugs.^25^^,^^29^^,^^30^

#### WHO-Multidrug therapy (MDT/WHO)

In the MDT/WHO group, twenty multibacillary patients were enrolled and treated with the standard MDT/WHO regimen, which consisted of a 3-drug regimen of rifampicin (600 mg monthly), dapsone (100 mg once a month and daily), and clofazimine (300 mg monthly and 50 mg daily). The treatment duration was 6-months for PB patients and 12-months for MB patients.

### Statistical analysis

All data were analyzed with GraphPad Prism v.9.0 software (GraphPad Inc., La Jolla, CA, USA). The Mann-Whitney test analyzed statistical differences for comparison among diverse groups, and the Wilcoxon Test was used to compare different times in the same treatment during the follow-up. Furthermore, the Chi-Square test was employed to analyze contingency data regarding the presence or absence of symptoms across various groups. In instances where the expected frequencies were too low for the reliable application of the Chi-Square test, Fisher's exact test was utilized to assess the evolution of symptoms over the analyzed period. This approach ensured the accuracy of statistical comparisons even with small sample sizes. The Binomial Logistic Regression Analysis was performed in order to assess the association between age, sex, clinical symptoms evolution time for Hansen’s disease, and comorbidities (hypertension, type 2 diabetes, dyslipidemia, autoimmune diseases, and obesity) with the outcome therapeutic scheme (MDT/WHO: 0 and RIMOXCLAMIN: 1 Using the *jamovi* project (2021). *jamovi* (Version 1.6) [Computer Software]. Retrieved from https://www.jamovi.org. The data were expressed in Odds rRatio (OR) and 95 % Confidence iInterval (95 % CI).

## Results

The demographic and clinical data of the individuals in the RIMOXCLAMIN and MDT/WHO groups are described in Table 2. There were no significant differences between the groups regarding gender, age, and the clinical form of HD Regarding occupation, in the RIMOXCLAMIN group, 54.3 % corresponded to people in business and physicians, while in the PQT/WHO group, 85 % were retired. In both groups, we observed a lengthy period between the beginning of the symptoms and H.D. diagnosis, with a mean of 46-months for the RIMOXCLAMIN group and 40.2-months for the MDT/WHO group (*p* > 0.05).

**Table 2:** Demographic and clinical characterization of patients treated with RIMOXCLAMIN and MDT/WHO.

Characteristics		RIMOXCLAMIN patients (*n* = 46)		MDT/WHO patients (*n* = 20)		p-value
		n	%	n	%	
Sex	Male	30	65	10	50	0.37^a^
	Female	16	35	10	50	
Age (years)	Median (min ‒ max)	59.5 (16 – 88)		60 (10 – 78)		0.75^b^
Occupation	Businessperson	13	28.2	–	–	
	Physician	12	26.1	–	–	
	Teacher	4	8.7	–	–	
	Engineering	3	6.6	–	–	
	Laboratory Trade Representative	2	4.3	–	–	
	Student	–	–	3	15	
	Homeworker	–	–	3	15	
	Self-employed	–	–	7	35	
	Retired	12	26.1	7	35	
Clinical Classification	BB	39	84.8	16	80	0.12^c^
	PNL	6	13	1	5	
	BL	–	–	2	10	
	LL	1	2.2	1	5	
Symptoms (months)	Median (IQR)	36 (12 – 60)		24 (12 – 54)		0.74^b^

DT/WHO, WHO standard multidrug therapy; BB, Borderline-Borderline HD; PNL, Pure Neural HD; BL, Borderline-Lepromatous HD; LL, Llepromatous HD; IQR, Interquartile Ranger.

a Chi-Square with Yates' correction *t*-test.

b Unpaired Mann-Whitney *t*-test.

c Chi-Square t-test.

The logistic regression model was not significant (χ^2^ = 2.74; *p* = 0.949; *R*^2^ MacFadden = 0.0342; Accuracy = 0.708; Specificity = 0.05; Sensitivity = 1.0; Area Under the Curve [AUC = 0.607]), demonstrating an absence of association among symptoms evolution time [OR = 1.00 (95 % CI = 0.98‒1.02); *p* = 0.69], age [OR = 1.012 (95 % CI =0.97‒1.05); *p* = 0.48], sex [OR = 0.72 (95 % CI = 0.22‒2.31); *p* = 0.58], hypertension [OR = 0.81 (95 % CI = 0.14‒4.48); *p* = 0.81], type 2 diabetes [OR = 3.53 (95 % CI=0.29‒41.98); *p* = 0.31], dyslipidemia [OR = 0.73 (95 % CI = 0.10‒5.17); *p* = 0.75], autoimmune diseases [OR = 1.04 (95 %CI = 0.07‒13.89); *p* = 0.97], and obesity [OR = 0.99 (95 % CI = 0.19‒5.10); *p* = 0.99] with the clinical outcome (therapeutic scheme). The statistical report is in Supplementary File 1.

Clinical data at HD diagnosis for the RIMOXCLAMIN and the MDT/WHO groups are described in Table 3. We observed that in the RIMOXCLAMIN group, 56 % of patients had up to 5 skin lesions, and 44 % had more than five lesions, similar to the MDT/WHO group with 65 % and35 %, respectively. Regarding the affected nerves, the two groups showed extensive neural involvement for all analyzed parameters for patients with up to 5 and those with >5 skin lesions.

**Table 3:** Number of skin lesions, characterization of neurological symptoms, and quantification of altered points on esthesiometry of hands and feet of HD patients treated with RIMOXCLAMIN and MDT/WHO at HD diagnosis.

Number of skin lesions		0–5	> 5	p-value^c^
Number of patients	RIMOXCLAMIN (*n* = 46)	26	20	0.71
	MDT/WHO (*n* = 20)	13	7	
Average number of impaired nerves^a^	RIMOXCLAMIN (*n* = 46)	4.0	4.0	0.79
	MDT/WHO (*n* = 20)	6.0	6.9	0.33
Number of patients with pain on nerve palpation (percentages in parentheses)	RIMOXCLAMIN (*n* = 30)	17 (65.4)	13 (65)	0.78
	MDT/WHO (*n* = 9)	6 (46.1)	3 (42.9)	0.74
Number of patients with burning, prickling, stinging (percentages in parentheses)	RIMOXCLAMIN (*n* = 46)	25 (96.2)	20 (100)	0.89
	MDT/WHO (*n* = 20)	13 (100)	7 (100)	1.00
Number of patients with weakness (percentages in parentheses)	RIMOXCLAMIN (*n* = 46)	14 (53.8)	16 (80)	0.12
	MDT/WHO (*n* = 14)	9 (69.2)	5 (71.4)	1.00
Median altered esthesiometric points^b^	RIMOXCLAMIN (*n* = 46)	9.1	6	0.79
	MDT/WHO (*n* = 20)	12.6	20	0.69

a Impaired nerves: involvement detected by nerve palpation, neurophysiologic tests, and/or ultrasonography.

b Esthesiometric points by Semmes-Weinstein Monofilaments.

c Fisher's exact *t*-test.

Table 4 details the frequencies of affected nerves by palpation, electroneuromyography, and/or nerve USG at some point during the treatment. In both groups, the nerves most frequently affected were in the lower limbs compared to the upper limbs.

**Table 4:** Number and percentage of affected nerves (defined as abnormalities at least in one of three exams: palpation, ENMG, and USG) in HD patients before the treatment with RIMOXCLAMIN and MDT/WHO groups.

		Affected nerves (palpation, ENMG, USG)									
		Upper limbs						Lower limbs			
		Ulnar		Radial		Median		Fibular		Tibial	
		R	L	R	L	R	L	R	L	R	L
**RIMOXCLAMIN**	n	25	33	6	6	5	4	29	26	26	25
	%	54	72	13	13	11	9	63	57	57	54
**MDT/WHO**	n	15	11	3	3	13	15	15	18	19	14
	%	75	55	15	15	65	75	75	90	95	70
**Sum of both groups**	n	40	44	9	9	18	19	44	44	45	39
	%	61	67	14	14	27	29	67	67	68	59

ENMG, Electroneuromyography; USG, Peripheral nerve Ultrasonography; R, Right; L, Left.

Data on the pattern of neural involvement and the respective Physical Disability Grade (PDG) in the RIMOXCLAMIN and MDT/WHO groups, at baseline and after 3, 6, and 12-months of treatment, are described in Table 5. Regarding nerve palpation, we observed a reduction in the frequency of thickening in both groups: in the RIMOXCLAMIN group, the frequency decreased from 65 % at baseline to 28 % after 6 months of treatment (*p* = 0.03) and further to 9 % after 12 months (*p* = 0.03); in the MDT/WHO group, it decreased from 95 % to 90 % after 6-months of treatment (*p* > 0.99) and then to 40 % after 12-months (*p* = 0.002). The greatest difference between the groups was observed at the 6-month follow-up visit (*p* < 0.0001). Nerve pain on palpation also showed a reduction in frequency in both groups when comparing baseline to 12-months of treatment. A statistically significant difference was observed by the 6th month in both groups (*p* = 0.0003 for RIMOXCLAMIN and *p* = 0.0310 for MDT/WHO).

**Table 5:** Number of patients with one or more thickened and/or painful nerves on palpation and Physical Disability Grade (PDG) at baseline and during follow-up for RIMOXCLAMIN and MDT/WHO groups.

		Initial		3^r^^d^ month		6th month		12th month		p-value^b^ (follow-up)				
Follow-up										Initial to 3^r^^d^ month	3^r^^d^ to 6th month	Initial to 6th month	6th to 12th month	Initial to 12th month
Nerve on palpation		n	%	n	%	n	%	n	%					
≥1 Thickened	RIMOXCLAMIN	30	65.2	24	52	13	28	4	9	0.29	0.033	0.0007	0.03	<0.0001
	MDT/WHO	19	95	18	90	18	90	8	40	1	1	1	0.002	0.0004
	p-value/groups^a^	0.03		0.01		<0.0001		0.01						
≥1 Painful	RIMOXCLAMIN	30	65.2	22	48	12	26	4	9	0.14	0.05	0.0003	0.052	<0.0001
	MDT/WHO	9	45	8	40	2	10	0	0	1	0.06	0.0310	0.49	0.0012
	p-value/groups^a^	0.21		0.75		0.25		0.42						
PDG^c^										p-value^d^ (follow-up)				
PDG^c^ 0	RIMOXCLAMIN	13	28	20	43	27	59	36	78	0.19	0.21	0.006	0.072	<0.0001
	MDT/WHO	6	30	6	30	9	45	13	65	1	0.51	0.51	0.34	0.0562
	p-value/groups^d^	0.88		0.45		0.45		0.41						
PDG^c^ 1	RIMOXCLAMIN	21	46	18	39	15	32	9	20	0.67	0.51	0.197	0.22	0.0065
	MDT/WHO	13	65	12	60	10	50	6	30	1	0.75	0.52	0.33	0.0562
	p-value/groups^d^	0.24		0.195		0.215		0.41						
PDG^c^ 2	RIMOXCLAMIN	12	26	8	18	4	9	1	2	0.3	0.52	0.052	0.36	0.0017
	MDT/WHO	1	5	2	10	1	5	1	5	1	1	1	1	1
	p-value/groups^d^	0.1		0.86		0.988		0.87						

a Unpaired Mann-Whitney *t*-test.

b Paired Wilcoxon matched-pair *t*-test.

c Physical Grade Disability.

d Fisher's exact *t*-test.

Regarding the degree of physical disability, considering the distribution between absence of disability (grade zero) and presence of disability (degrees 1 + 2) over time, we observed a reduction in the degree of disability with RIMOXCLAMIN treatment, with an increase in the number of patients with PDG 0 initially from 13 to 27 in the 6th month of treatment (*p* = 0.006) and 36 in the 12th month of treatment (*p* < 0.0001). In the MDT/WHO group, we also observed an increase in the number of patients with PGD 0 from 6 to 13 at the end of treatment, but no significant difference was found between times (*p* > 0.05). However, when we compared both groups at each assessment time, we did not find significant difference between the number of patients and their PDG.

Data on neurological symptoms during follow-up in the RIMOXCLAMIN and MDT/WHO groups are described in Table 6. The significant reduction in all neurological symptoms, except weakness, is noteworthy in the third month of treatment with RIMOXCLAMIN, a fact not found for any symptom in the MDT/WHO group. After 6-months of treatment, we observed significantly reduced numbness and tingling in both groups. At the end of treatment with RIMOXCLAMIN, the number of patients with numbness was significantly lower than in the MDT/WHO group (*p* = 0.02). Regarding the symptoms of tingling, cramps, and weakness, it should be noted that both regimens showed significant efficacy only after 12-months of treatment (*p* < 0.05).

**Table 6:** Number of patients according to the presence of neurological symptoms: numbness, tingling, cramping, pain symptoms (burning, pins, burning), weakness, and physical grade disability, at baseline and during the follow-up of treatment with RIMOXCLAMIN and MDT/WHO regimens.

Follow-up		Initial		3^r^^d^ month^c^			6th month^c^			12th month^d^		
Symptoms		n	%	n	%	p-value^b^ (initial to 3^r^^d^ month)	n	%	p-value^b^ (3^r^^d^ to 6th month)	n	%	p-value^b^ (6th to 12th month)
Numbness	RIMOXCLAMIN	44	95.7	37	86	0.025	24	55.8	0.008	12	26.1	0.02
	MDT/WHO	20	100	20	100	1	15	75	0.047	12	60	0.5
	p-value/groups^a^	0.87		0.07		–	0.14		–	0.02		–
Tingling	RIMOXCLAMIN	44	95.7	36	83.7	0.03	18	41.9	0.0003	6	13	0.008
	MDT/WHO	20	100	20	100	1	15	75	0.047	4	20	0.001
	p-value/groups^a^	0.87		0.06		–	0.016		–	0.73		–
Cramps	RIMOXCLAMIN	28	60.9	15	34.9	0.012	9	20.9	0.237	3	6.5	0.07
	MDT/WHO	20	100	16	80	0.10	13	65	0.48	4	20	0.001
	p-value/groups^a^	0.003		0.001		–	0.001		–	0.23		–
Pain (burning, pickling, stinging)	RIMOXCLAMIN	36	78.3	24	55.8	0.015	16	37.2	0.14	9	19.6	0.16
	MDT/WHO	14	70	12	60	0.74	2	10	0.002	0	0	0.49
	p-value/groups^a^	0.68		0.75		–	0.08		–	0.08		–
Weakness	RIMOXCLAMIN	26	56.5	20	46.5	0.3	13	30.2	0.19	2	4.3	0.004
	MDT/WHO	14	70	14	70	1	13	65	1	2	10	0.0008
	p-value/groups^a^	0.45		0.09		–	0.01		–	0.75		–

a Unpaired Mann-Whitney *t*-test.

b Paired Wilcoxon matched-pair *t*-test.

c Data not available for 3 patients (RIMOXCLAMIN).

d Data not available for 1 patient (RIMOXCLAMIN).

Cramps were initially reported by all patients in the MDT/WHO group and by 60.9 % of those in the RIMOXCLAMIN group, making this the only symptom with a significant baseline difference between groups. During follow-up, the relative reduction in cramps was greater in the RIMOXCLAMIN group at all time points. After three months of treatment, a 42.7 % reduction was observed in the RIMOXCLAMIN group compared to 20 % in the MDT-WHO group. At six months, these reductions reached 60.3 % and 35 %, respectively. By the end of treatment, both groups showed comparable results, with an 89.3 % reduction in the RIMOXCLAMIN group and 80 % in the MDT-WHO group, demonstrating a similar final outcome despite initial differences.

The results for the pain scale during follow-up are described in Table 7. Initially, most patients had a pain scale ≥ 3, 80.4 % in the RIMOXCLAMIN group and 95 % in the MDT/WHO group (*p* = 0.14). After 3-months, there was a significant reduction in the frequency of patients with a pain scale ≥ 3 in the RIMOXCLAMIN group to 47.8 %, while the MDT/WHO group was almost unchanged (*p* = 0.004). At 6 and 12 months, both groups showed significant improvement of pain.

**Table 7:** Distribution of patients according to the Pain Scale at the beginning and during follow-up of treatments with RIMOXCLAMIN and MDT/WHO.

Follow-up		Initial		3rd month		6th month		12th month	
		p-value of time^2^		RIMOXCLAMIN	<0.0001	<0.0001		<0.0001	
Pain scale				MDT¹	0.07	0.0001		0.0049	
		n	%	n	%	n	%	n	%
0 – 2	RIMOXCLAMIN	9	19.6	24	52.2	39	84.4	42	91.3
	MDT/WHO	2	10	3	15	9	45	12	60
3 – 5	RIMOXCLAMIN	25	54.3	18	39.1	6	13	4	8.7
	MDT/WHO	11	55	8	40	7	35	8	40
> 5	RIMOXCLAMIN	12	26.1	4	8.7	1	2.2	–	–
	MDT/WHO	7	35	9	45	4	20	–	–
p-value^1^ (comparison between treatment regimens)		0.14		0.004^3^		0.17^4^		0.06^5^	

1 Unpaired Mann-Whitney *t*-test.

2 Paired Wilcoxon matched-pair *t*-test.

3 Data not available for 3 patients (RIMOXCLAMIN).

4 Data not available for 4 patients (RIMOXCLAMIN).

5 Data not available for 2 patients (RIMOXCLAMIN).

Data on complementary tests performed by patients at any time during the follow-up (diagnosis and during treatment) in the RIMOXCLAMIN and MDT/WHO groups are described in Table 8. All patients from both groups underwent at least one laboratory test. In the RIMOXCLAMIN group, 14 patients (30.4 %) had one positive laboratory test, while in the MDT/WHO, 9 (45 %) patients had at least one positive laboratory test. Among 46 patients who underwent USG in the RIMOXCLAMIN group, 43 (93.5 %) confirmed Multiple Mononeuropathies (MPNM), while in the MDT/WHO, this number was 20 (100 %). In the RIMOXCLAMIN group, 43 ENMG were performed, with MPNM in 35 (81.4 %), while in the MDT/WHO group, 5 tests were performed, with MPNM in 4 (80 %). Considering both USG and ENMG results, 100 % of the RIMOXCLAMIN group had abnormalities in at least one of the tests, and 32 (69.6 %) had abnormalities in both.

**Table 8:** Complementary test results for patients treated with RIMOXCLAMIN and MDT/WHO.

Complementary Exams		RIMOXCLAMIN group (*n* = 46)		MDT/WHO group (*n* = 20)		Sum of both groups (*n* = 66)	
		n	%	n	%	n	%
RLEP-PCR	Realized	Z		16		56	
	Positive	10	25	9	56.25	19	28.6
	Negative	27	67.5	6	37.5	33	64.3
	Inconclusive	3	7.5	1	6.25	4	7.1
ELISA aPGL-I	Realized	44		16		60	
	Positive	7	16	6	37.5	13	21.7
	Average Index	1.74		3.3		2.7	
	Negative	37	84	10	62.5	47	78.3
Bacilloscopy	Realized	5		8		13	
	Positive	1	20	4	50	5	38.5
	Average BI^f^	6		3.5		4	
	Negative	4	80	4	50	8	61.5
Laboratorials^a^	At least 1 performed	46		20		66	
	≥1 positive test	14	30.4	9	45	23	34.9
	≥2 positive tests	4	8.7	4	20	8	12.1
	3 positive tests	1	2.2	3	15	4	6.1
USG-NP^b^	Realized	46		20		66	
	Normal	3	6.5	0	0	3	4.5
	MNPM^c^	43	93.5	20	100	63	95.5
	Other diseases	0	0	0	0	0	0
ENMG^4^	Realized	43		5		48	
	Normal	4	9.3	0	0	4	8.3
	MNPM^c^	35	81.4	4	80	39	81.3
	Other diseases	4	9.3	1	20	5	10.4
USG-NP^b^ and/or ENMG^d^	Normal in both	0	0	0		0	
	MNPM^c^ in 1	46	100	20	100	66	100
	MNPM^c^ in both^e^	32	69.6	4	20	36	54.5

a Laboratory: RLEP-PCR, ELISA aPGL-I, and slit skin smear microscopy.

b USG, Ultrasonography of Peripheral Nerves.

c MNPM, Multiple Mononeuropathy.

d ENMG, Electroneuromyography.

e Both: USG-PN and ENMG; It is unreliable to perform statistical analysis for this parameter since 27 % of the patient sample did not undergo ENMG.

f IB, Bacilloscopic index.

The tactile sensory evaluations of the hands and feet using the SWM in the RIMOXCLAMIN and MDT/WHO groups during follow-up are described in Table 9. In the RIMOXCLAMIN group, at the end of treatment, there was a 0.5 % reduction in the number of abnormal points on the hands compared to the beginning of treatment, while in the MDT/WHO group, there was a 5.4 % increase of abnormal points. In the feet, the RIMOXCLAMIN group showed a reduction of 17.9 % of altered points, while in the MDT/WHO group, there was a reduction of 10.3 %.

**Table 9:** Percentage of hand and foot sensory points using Semmes-Weinstein Monofilaments at baseline and after treatment in RIMOXCLAMIN and MDT/WHO groups.

Time	HANDS (%)				FEET (%)			
SMW	Before		After		Before		After	
	RIMOXCLAMIN	MDT/WHO	RIMOXCLAMIN	MDT/WHO	RIMOXCLAMIN	MDT/WHO	RIMOXCLAMIN	MDT/WHO
Green (0.07gf)	92.7	79.3	93.2	73.9	43.4	37.0	72.3	35.5
Blue (0.20gf)	3.7	8.9	4.7	17.5	23.2	15.0	12.2	26.8
Violet (2.00gf)	1.9	11.8	1.5	8.2	17.6	30.9	9.3	26.4
Red (4.00gf)	1.1	0	0.6	0.4	7.8	8.4	3.4	5.7
Orange (10.0gf)	0.2	0	0	0	3.1	5.5	1.8	2.0
Pink (300gf)	0.2	0	0	0	3.1	2.7	0.8	3.0
Black (>300gf)	0.3	0	0	0	1.8	0.5	0.2	0.7
Normal	92.7	79.3	93.2	73.9	66.6	52.0	84.5	62.3
altered	7.3	20.7	6.8	26.1	33.4	48.0	15.5	37.7

SWM, Semmes-Weinstein Monofilaments.

**Fig. 1:**
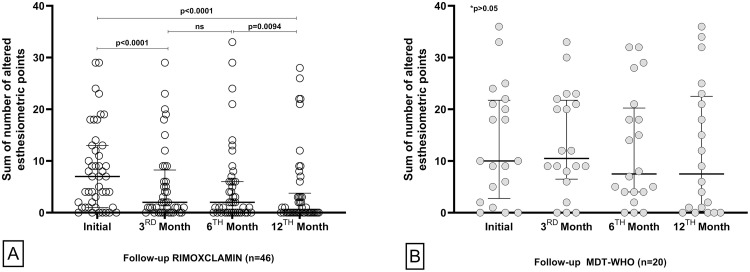
Distribution of the sum of altered esthesiometric points in hands and feet per patient during the follow-up of RIMOXCLAMIN (A) and MDT/WHO (B) groups. The Wilcoxon matched-pairs test determined statistical significance. ns: no significance. Data not available for 6 patients (3rd month ‒ RIMOXCLAMIN), 4 patients (6th month ‒ RIMOXCLAMIN), and 2 patients (12th month ‒ RIMOXCLAMIN).

In Fig. 1A, in the RIMOXCLAMIN group, a significant difference was identified between the initial [Median: 7.0 (IQR: 1.0‒13.0)] and the 3rd month of treatment [Median: 2.0 (IQR: 0.0‒8.25); *p* < 0.0001]. This difference was not observed at 6th month [Median: 2.0 (IQR: 0.0‒6.0); *p* = 0.68]. However, in relation to the 6th month of treatment, in the 12th month, a statistical difference was observed [Median: 0.0 (IQR: 0.0‒3.75); *p* = 0.0094]. In Fig. 1B, in the MDT/WHO group, no statistical difference was identified between any of the times evaluated: between the initial time [Median: 10.0 (IQR: 2.75‒21.75)] and the third month of treatment [Median: 10.5 (IQR: 6.5‒21.75); *p* = 0.90] between the 3rd and 6th month [Median: 7.5 (IQR: 4.0‒20.25); *p* = 0.97]; between the 6th month and the end of treatment [Median: 7.5 (IQR: 0.25‒22.5); *p* = 0.71]. Regarding the baseline, no statistical difference was observed as compared to the 12th month (*p* = 0.81).

**Fig. 2:**
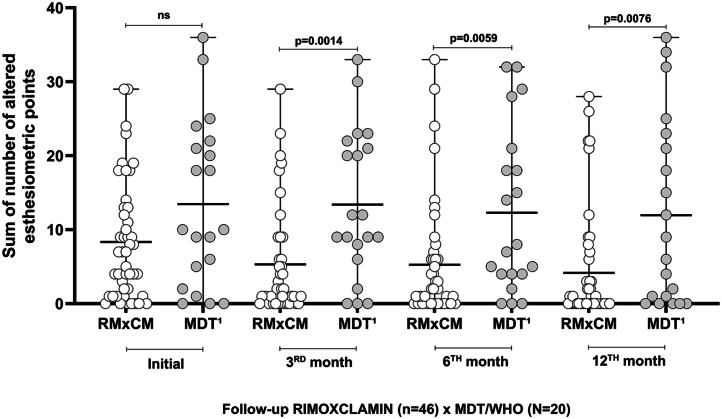
Comparative distribution of the sum of altered esthesiometric points in hands and feet per patient during the follow-up of RIMOXCLAMIN and MDT/WHO groups. The Mann-Whitney unpaired test determined statistical significance. ns: no significance (*p* = 0.09). Data not available for 6 patients (3rd month ‒ RIMOXCLAMIN), 4 patients (6th month ‒ RIMOXCLAMIN), and 2 patients (12th month ‒ RIMOXCLAMIN).

In Fig. 2, at the beginning of treatment, there was similarity between the RIMOXCLAMIN group [Median: 7.0 (IQR: 1.0‒13.0)] and the MDT/WHO group [Median: 10.0 (IQR: 2.75‒21.75); *p* = 0.09]. At the end of the 3rd month of treatment, we found a significant reduction in the sum of the altered points in the RIMOXCLAMIN group [Median: 2.0 (IQR: 0.0‒8.25)], as compared to the MDT/WHO group [Median: 10.5 (IQR: 6.5‒21.75); *p* = 0.014]. In 6th-month, the difference was maintained (*p* = 0.0059) between the RIMOXCLAMIN group [Median: 2.0 (IQR: 0.0‒6.0)] and the MDT/WHO group [Median: 7.5 (IQR: 4‒20.25)]. At the 12th month of treatment, the RIMOXCLAMIN group [Median: 0.0 (IQR: 0.0‒3.75)] remained different in outcome from the MDT/WHO group [Median: 7.5 (IQR: 0.25‒22.50); *p* = 0.0076].

Adverse reactions were more frequent in the MDT/WHO than in the RIMOXCLAMIN group. 17 (85 %) patients of the MDT/WHO group reported any adverse effect compared to 24 (52 %) patients of the RIMOXCLAMIN group (*p* = 0.0244). Table 10 demonstrates the frequency of specific symptoms. Symptoms of abdominal pain, weakness, fatigue, headache, and epigastric pain were significantly more frequent in the MDT/WHO group than in the RIMOXCLAMIN group. All adverse effects in the RIMOXCLAMIN group were mild, and no one patient had to discontinue treatment, while in MDT/WHO, seven patients had changed their treatment mainly because of symptoms of anemia.

**Table 10:** Adverse effects during the treatment with RIMOXCLAMIN and MDT/WHO.

Adverse effect	RIMOXCLAMIN		Average time	MDT/WHO		Average time (months)	p-value^a^
	n	%		n	%		
Muscle pain	0		0	1	5	1	0.126
Dyspnea	0		0	1	5	2	0.126
Dizziness	0		0	1	5	6	0.126
Vertigo	0		0	1	5	3	0.126
Hyporexia	0		0	1	5	0	0.126
Change in Heart Rhythm	1	2.2	4	0	0	0	0.51
Conjunctivitis	1	2.2	7	1	5	6	0.54
Nausea and vomiting	2	4.3	1	1	5	4	0.91
Constipation	3	6.5	1	1	5	4	0.81
Balance change	4	8.7	4	1	5	1	0.60
Ichthyosis	6	13.0	5	3	15	4	0.83
Bitter taste	7	15.2	3	2	10	5	0.57
Diarrhea	5	10.9	1	2	10	1	0.92
Hyperchromia	10	21.7	7	3	15	4	0.52
Abdominal pain	0		0	2	10	2	0.03
Malaise	2	4.3	1	5	25	3	0.012
Headache	0		0	5	25	2	0.0004
Epigastric pain	1	2.2	6	7	35	4	0.0002
Fatigue	1	2.2	6	8	40	3	<0.0001

a Chi-square *t*-test.

## Discussion

This is the first study investigating the clinical response in neurological signs and symptoms for a new multidrug regimen to treat HD, the RIMOXCLAMIN. In this study, we compared the neurological and clinical evaluations between the standard HD treatment, the MDT/WHO, and the RIMOXCLAMIN.

Despite the reliance on the number of skin lesions for the operational classification of leprosy into paucibacillary and multibacillary forms, our data reveal that this parameter alone may not adequately reflect the extent of neural involvement. In both the RIMOXCLAMIN and MDT/WHO groups, we observed comparable distributions of patients with ≤ 5 and > 5 skin lesions. However, across both subgroups, patients exhibited significant neural impairment regardless of skin lesion count. This finding underscores the clinical heterogeneity of leprosy and highlights the limitation of using lesion count as a sole indicator of disease severity.

Furthermore, the observation that patients with few skin lesions may still present extensive neural damage reinforces the need to revise the criteria used for disease classification and treatment decisions. An overreliance on skin lesion count risks underestimating disease severity, particularly in individuals with predominant neural involvement but limited cutaneous manifestations. Therefore, comprehensive clinical and neurological assessments should be prioritized to ensure accurate diagnosis, appropriate classification, and optimized therapeutic strategies. These findings support the argument for integrating more sensitive neurological parameters into operational guidelines for leprosy diagnostic and management.

We found that RIMOXCLAMIN was significantly quicker and more effective than MDT/WHO in improving tactile sensory tests in hands and feet. Considering that HD neuropathy is responsible for disabilities and deformities,^43^ we advocate that neurological evaluation is a vital strategy to evaluate response to the standard and new therapeutic regimens for HD.

Both groups, RIMOXCLAMIN and MDT/WHO, were similar at baseline considering demographic, clinical, and laboratory characteristics. The demographic characteristics of the studied sample are in accordance with epidemiological data for Brazil: analyses of new cases detected between 2015 and 2019 showed that 55.3 % of patients were male, and the highest new case detection rate occurred among people above five years old in both genders.^44^ On the other hand, 75 % of the subjects in the RIMOXCLAMIN group had high-income occupations, diverging data on Neglected Tropical Diseases (NTDs), including HD, is strongly associated with poverty and inequality. This finding can be explained because, in the RIMOXCLAMIN group, patients financed their treatments themselves due to the lack of availability of substitutive antibiotics in the public health system and because there was no public or private funding for this research. Considering the binomial logistic regression analysis, we emphasize that demographic characteristics, clinical symptoms evolution time, and comorbidities were not confounding agents in the clinical and neurological analyses due to the lack of difference between the MDT/WHO and RIMOXCLAMIN groups for these variables.

Regarding the clinical forms, both groups showed a predominance of borderline-borderline forms (above 80 %), corroborating with published papers from our group that studied samples in the same region with medium or high endemicity, considering the prevalence rates in the last 10-years. This result highlights an expressive frequency of new cases as borderline HD with a hypochromic macular clinical presentation.^1^, ^2^, ^3^^,^^5^, ^6^, ^7^, ^8^^,^^10^^,^^11^

The similarity between the groups was also demonstrated by the long time between the beginning of the symptoms and the diagnosis, with medians of 36 months in the RIMOXCLAMIN group and 24 months in the MDT/WHO group, highlighting the diagnostic delay for the clinical forms characterized essentially by neurological manifestations, with mild dermatological manifestations.

A relevant result was the lack of correlation between the number of skin lesions and the neural involvement, expressed by the average number of affected nerves, pain symptoms, weakness, and altered points on esthesiometry. This result is reinforced by the observation that the patients in both groups mostly presented with less than five skin lesions despite the high frequencies of neurological abnormalities detected at baseline evaluation.

Regarding the median of altered esthesiometric points on hands and feet, independent of the number of skin lesions, we found a mean of 2.5 altered points in the RIMOXCLAMIN group and 5 points in the MDT/WHO group; furthermore, all patients in both groups had at least 2 altered points at the baseline evaluation. The RIMOXCLAMIN group had 7.3 % and 33.4 % altered points on hands and feet, respectively, while the MDT/WHO group showed higher frequencies of altered points (20.7 % and 48 %, respectively). These results reinforce the predominance of lower limb impairment by H.D. compared to upper limbs. These results for both groups were lower than those previously published by Frade et al. (2022) in a study that evaluated 107 HD patients by tactile sensation using the SWM at diagnosis, in which the authors found 43 % of the SWM-test points altered on the hands and 87.9 % on the feet.^13^

Among the laboratory tests (bacilloscopy, RLEP-qPCR, and ELISA aPGL-I) from both studied groups, we observed a positivity rate of 34.9 % in at least one of them, with a higher positivity in the laboratory tests for the MDT/WHO group. These results demonstrate the limitations of these laboratory tests in confirming the HD diagnosis, considering their low sensitivity, which was presented in previously published papers from the same geographic region.^31^

On the other hand, neurological morphofunctional tests (i.e., neurophysiologic exams and nerve USG) proved to be more efficient in elucidating the diagnosis of HD since all patients had abnormalities in at least one of the tests. It is worth mentioning that USG of peripheral nerves showed signs of Peripheral Multiple Mononeuropathy in 93.5 % of patients from the RIMOXCLAMIN group and 100 % of the patients from the MDT/WHO group, while in both groups, around 81 % of patients also presented Peripheral Multiple Mononeuropathy.

According to the global guidelines, Brazil uses only one criterion for release from HD treatment: the completion of standard treatment with MDT/WHO for 12 months for multibacillary patients.^12^ The physical impairment associated with HD is usually secondary to nerve damage resulting from chronic granulomatous inflammation due to *Mycobacterium leprae*. Multidrug Treatment (MDT) can cure HD. if instituted at an early stage, thus could prevent disability.^45^

Regarding the effectiveness of the two multidrug regimens, measured by the frequency of neurological symptoms, in the third month, the RIMOXCLAMIN group showed a significant reduction of 20 % in numbness, tingling, and pain, 47 % in cramps and 33 % in weakness, higher values than observed in the MDT/WHO group, that showed no reduction for numbness, tingling and weakness, and only 20 % reduction for cramps and 14 % for pain. In the sixth and twelfth months, the reduction in the percentages of numbness and tingling were greater in the RIMOXCLAMIN group than in the MDT/WHO group. Regarding recovery from weakness, the RIMOXCLAMIN group was 26 % better than MDT/WHO, with only 5 % in the sixth month and similar results above 52 % in the final assessment. The significant difference in the prevalence of cramps at baseline suggests that the initial clinical status of patients in the MDT-WHO group may have been more severe regarding this specific symptom. However, the faster symptom relief in the RIMOXCLAMIN group indicates a potential advantage of this regimen in accelerating neurological recovery. While both treatments ultimately led to similar final outcomes, the earlier improvement observed with RIMOXCLAMIN may have relevant implications for patient quality of life, particularly in reducing discomfort and functional impairment during treatment. These findings reinforce the need to consider not only absolute efficacy but also the speed of symptomatic relief when evaluating new therapeutic strategies for HD.

Furthermore, the analysis of the SWM results also demonstrated that the RIMOXCLAMIN group achieved significant improvements earlier, with a significant reduction in the number of altered points as early as the third month of treatment, which showed progressive reductions until the last evaluation. In the MDT/WHO group, however, the number of altered hand and foot points did not show any improvement over time. In other words, the RIMOXCLAMIN regimen was significantly more effective than the MDT/WHO at all follow-up assessment times.

Specifically, the RIMOXCLAMIN treatment decreased the number of altered points on the hands by 0.5 % while the MDT/OMS treatment increased it by 5.4 %, and in relation to the altered points on the feet, the RIMOXCLAMIN treatment decreased 17.9 % while the MDT/OMS treatment only 10.3 %. In the literature, only our group has published about the patient follow-up considering the number of altered neural points using the SWM-test, and similar low results after one year of multibacillary multidrug therapy were found because the authors described the decreasing percentage of patients with altered SWM-test to 18 % for the hands, and to 28.7 % for the feet.^13^

Several authors have reported that the MDT/WHO does not result in recovery from neural damage by only measuring the evolution of the Physical Disability Grade (PDG) after release treatment.^46^, ^47^, ^48^ Our results demonstrated the same in relation to MDT/WHO. However, on the other hand, this seems to be related to the different efficacy of multidrug therapy, as the efficacy achieved with RIMOXCLAMIN treatment through the rapid and significant reduction of neurological symptoms, changes esthesiometric points by SWM, in addition to the significant reduction of the number of patients with degree of physical disability higher than zero.

Regarding the adverse effects related to the two therapeutic regimens, although hyperchromia was a similar report in both groups, in the RIMOXCLAMIN group, it was due to minocycline, manifested by hyperpigmented macules on photoexposed areas, and not related to diffuse hyperpigmentation caused by clofazimine, observed in the MDT/WHO group, which is a known cause of stigmatization.^5^^,^^15^^,^^19^^,^^20^^,^^26^^,^^49^ Also noteworthy were the symptoms related to hemolytic anemia caused by dapsone in the MDT/WHO group (malaise and fatigue), corroborating the findings of our referral center in a sample of 258 patients, among whom almost 60 % males and 56 % females evolved with anemia in the first month of follow-up with MDT/WHO.^50^ These symptoms were much less frequent in the treatment with RIMOXCLAMIN epigastralgy, and headaches were significantly more frequent in the MDT/WHO group than in the RIMOXCLAMIN group.

The results reported in the present manuscript indicate that treatment with RIMOXCLAMIN may be superior in terms of quick recovery of neurological damage, evidenced by the significant improvement of symptoms and sensitivity in hands and feet (SWM) as early as the third month, with a progressive improvement that was maintained during the 6- and 12-months follow-up as compared to MDT/WHO treatment, which achieved this similar improvement only in the 12th month of follow-up.

This study had some limitations. The RIMOXCLAMIN group was mainly composed of subjects with higher-income occupations; it is possible that other socioeconomic factors, such as better nutrition, access to physiotherapy and rehabilitation, and availability of medical rest, could have interfered with better neurological outcomes. Additionally, we evaluated only clinical variables (nerve palpation, nerve pain, hand and feet esthesiometry, and physical disability grade). Further research should address microbiological, molecular, and serological analyses. Furthermore, studies with more patients and longer follow-ups are needed to confirm these observations and consolidate scientific evidence, especially regarding response to treatment and possible adverse effects. Also, new studies with patients with a high load of bacilli should be performed to know precisely about the bactericidal efficacy of the RIMOXCLAMIN regimen and duration, besides the risk of relapses, and health costs and financing. Thus, this work is a pilot and initial study exploring a new intervention and an innovative application to evaluate the efficacy of other multidrug regimens for HD. Prospective studies with a larger population will be necessary in the future. Consequently, the study showed other treatment alternatives with new possibilities for resistance conditions or cases where it is impossible to use drugs from the MDT/WHO scheme. The final result is compared with the standard treatment and quickly sensory recovery.

This first report serves as a starting point to support future investigations that can contribute to the optimization of HD treatment, aiming for earlier improvement in patient's quality of life, using more effective multidrug therapy with less adverse effects, and with significant recovery of sensitivity and reducing the physical disability grade directly collaborating with the WHO objectives regarding the interruption of bacillary transmission and effective control of the HD with less clinical damage to patients.

## Authors’ contributions

MACF contributed to the study's conception and design. MACF and GSA substantially contributed to the acquisition of data, and both HBL and FRL for the analysis and interpretation of data. MACF, HBL, FCP, A.W., FACF, NAP, and WMJ contributed to the clinical care of patients. MACF and GAS provided the acquisition of clinical data. NAP and FRL executed and interpreted the laboratory tests. MACF, GSA, FRL, and HBL contributed to the statistical analysis and interpretation of the data. MACF provided scientific guidance and advice. MACF approved the final submitted version and provided supervision and orientation of the study. All authors contributed to the interpretation of the results and critical revision.

## Funding

This study was financed in part by Coordenação de Aperfeiçoamento de Pessoal de Nível Superior-Brazil (CAPES) ‒ Finance Code 001; by Fundação de Amparo à Pesquisa do Estado de São Paulo (FAPESP): 2021/13429-1 and 2023/00589-6 for 10.13039/100012137MACF, and with Scholarships Program for GSA (2023/00636–4), ; National Council for Scientific and Technological Development (CNPq) with a research grant for MACF (423635/2018–2). Brazilian Health Ministry (MS/FAEPA-FMRP-USP: 749145/2010 and 767202/2011), and Oswaldo Cruz Foundation-Ribeirão Preto (TED 163/2019-Protocol n° 25380.102201/2019–62/Project Fiotec: PRES-009-FIO-20). We also acknowledge the financial support of the Research and Assistance Support Foundation of the Hospital of the Medical School of Ribeirão Preto at USP (FAEPA) to the National Referral Center for Sanitary Dermatology and H.D. The funders had no role in study design, data collection and analysis, decision to publish, or preparation of the manuscript.

## Conflicts of interest

The authors declare that the research was conducted in the absence of any commercial or financial relationships that could be construed as a potential conflict of interest.

## Appendix Supplementary materials

Image, application 1

Image, application 2

Image, application 3
