# Supplementary material for: RIMOXCLAMIN: New therapeutic regimen for Hansen’s Disease cure based on effective sensitivity recovery
Source: Braz J Infect Dis. 2025 May 14;29(4):104539. doi: 10.1016/j.bjid.2025.104539 (PMC12141834; doi:10.1016/j.bjid.2025.104539)
Supplement: Supplementary file 1 [file mmc1.docx]

**BJID-D-24-00366_ Supplementary Materials**

**Supplementary File 1** Statistical report.

**Supplementary File 2** Raw data.
