# Supplementary material for: RIMOXCLAMIN: New therapeutic regimen for Hansen’s Disease cure based on effective sensitivity recovery
Source: Braz J Infect Dis. 2025 May 14;29(4):104539. doi: 10.1016/j.bjid.2025.104539 (PMC12141834; doi:10.1016/j.bjid.2025.104539)
Supplement: Supplementary file 2 [file mmc2.pdf]

Results

Binomial Logistic Regression

Model Fit Measures

				Overall Model Test		
Model	Deviance	AIC	R^2_{McF}	χ^2	df	p
1	77.5	95.5	0.0342	2.74	8	0.949

Model Coefficients - Treatment

						95% Confidence Interval	
Predictor	Estimate	SE	Z	p	Odds ratio	Lower	Upper
Intercept	0.09604	0.98403	0.09760	0.922	1.101	0.1600	7.57
Time of evolution	0.00293	0.00750	0.39093	0.696	1.003	0.9883	1.02
Age	0.01217	0.01731	0.70301	0.482	1.012	0.9785	1.05
Sex:							
1 – 0	-0.32560	0.59366	-0.54846	0.583	0.722	0.2256	2.31
Hypertension:							
1 – 0	-0.19955	0.86697	-0.23017	0.818	0.819	0.1498	4.48
Type 2 Diabetes:							
1 – 0	1.26347	1.26209	1.00109	0.317	3.538	0.2981	41.98
Dyslipidemia:							
1 – 0	-0.30466	0.99388	-0.30654	0.759	0.737	0.1051	5.17
Autoimmune:							
1 – 0	0.04163	1.32106	0.03152	0.975	1.043	0.0783	13.89
Obesity:							
1 – 0	-0.00530	0.83368	-0.00635	0.995	0.995	0.1941	5.10

Note. Estimates represent the log odds of "Treatment = 1" vs. "Treatment = 0"

Prediction

Cut-Off Plot

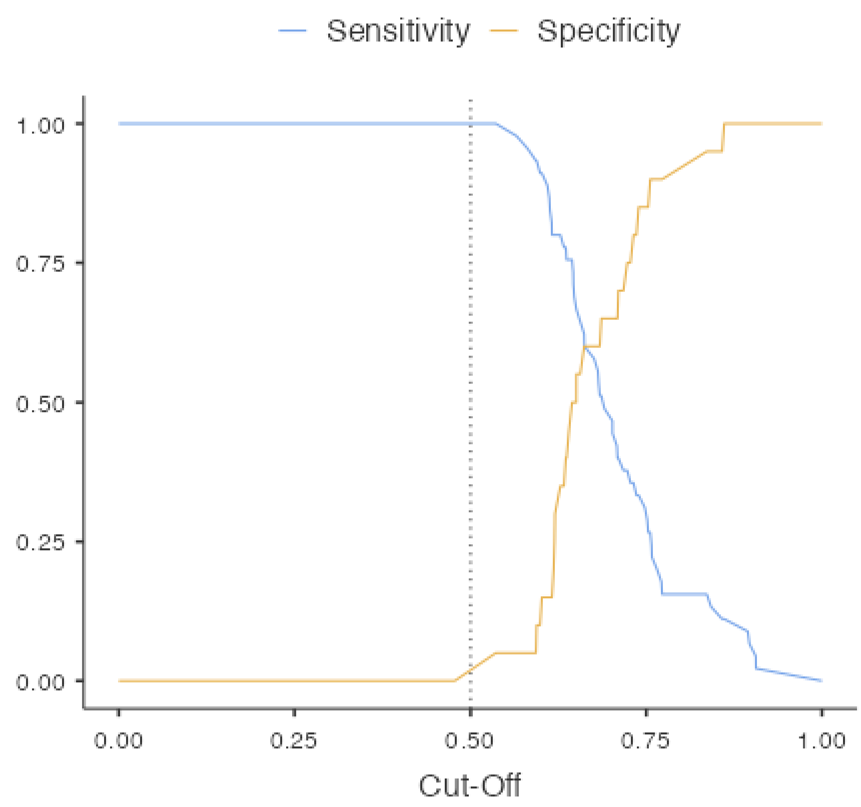

Classification Table – Treatment

	Predicted		
Observed	0	1	% Correct
0	1	19	5.00
1	0	45	100

Note. The cut-off value is set to 0.5

Predictive Measures

Accuracy	Specificity	Sensitivity	AUC
0.708	0.0500	1.00	0.607

Note. The cut-off value is set to 0.5

ROC Curve

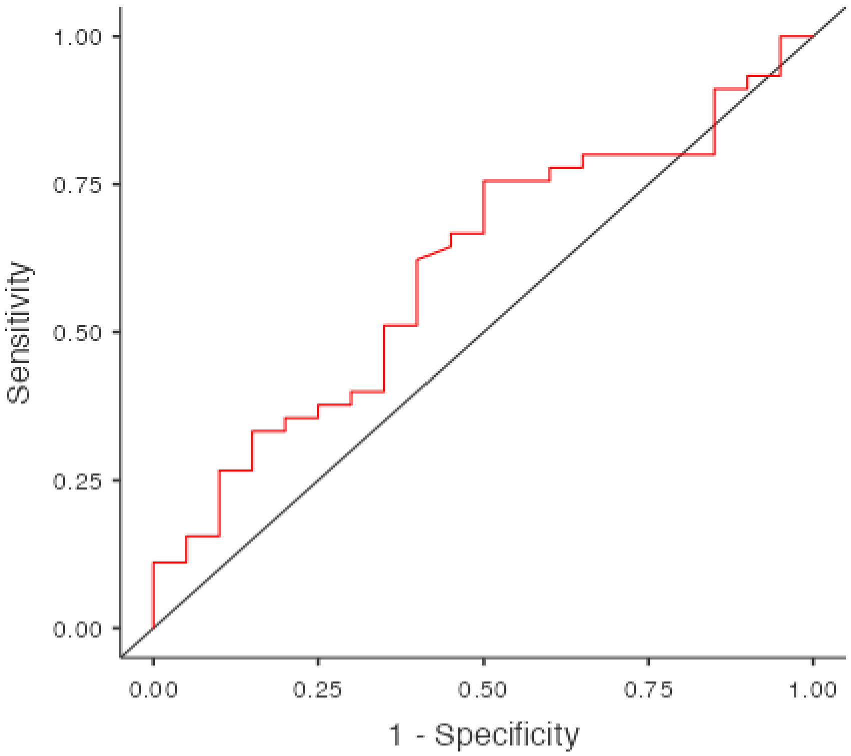

[3]

References

[1] The jamovi project (2021). *jamovi*. (Version 1.6) [Computer Software]. Retrieved from https://www.jamovi.org.

[2] R Core Team (2020). *R: A Language and environment for statistical computing*. (Version 4.0) [Computer software]. Retrieved from https://cran.r-project.org. (R packages retrieved from MRAN snapshot 2020-08-24).

[3] Sing, T., Sander, O., Beerenwinkel, N., & Lengauer, T. (2015). *ROCR: Visualizing the Performance of Scoring Classifiers*. [R package]. Retrieved from https://cran.r-project.org/package=ROCR.
